# Investigation on the mechanism of the combination of eremias multiocellata and cisplatin in reducing chemoresistance of gastric cancer based on *in vitro* and *in vivo* experiments

**DOI:** 10.18632/aging.205540

**Published:** 2024-02-09

**Authors:** Fan-e Cheng, Zheng Li, Xing Bai, Yanyan Jing, Junfei Zhang, Xiaoqian Shi, Tingting Li, Weiqiang Li

**Affiliations:** 1 Key Laboratory of Ningxia Minority Medicine Modernization Ministry of Education, Ningxia Medical University, Yinchuan 750004, Ningxia Hui Autonomous Region, China; 2 School of Traditional Chinese Medicine, Ningxia Medical University, Yinchuan 750004, Ningxia, China; 3 School of Basic Medicine, Zhejiang University of Chinese Medicine, Hangzhou 310053, Zhejiang, China; 4 Graduate School, Tianjin University of Chinese Medicine, Tianjin 300193, Tianjin, China

**Keywords:** chemotherapy resistance to gastric cancer, eremias multiocellata (EM), cisplatin (DDP), *in vivo* and *in vitro* experiments

## Abstract

Background: Cisplatin (DDP) is one of the important chemotherapy drugs for patients with advanced gastric cancer and metastasis, but its resistance is a bottleneck problem that affects clinical efficacy and patient survival. Eremias multiocellata (EM) is a traditional Chinese herbal medicine, which has been used in the treatment of precancerous lesions, gastric cancer, liver fibrosis, and other digestive diseases. However, the mechanism of reducing chemotherapy resistance to gastric cancer is still unclear.

Methods: We used the MTT assay to evaluate the proliferative viability of gastric cancer parental cell line MKN45 and its drug-resistant cell line MKN45/DDP, and compared their drug-resistance indices. The migration and invasion abilities of MKN45/DDP drug-resistant cells were evaluated using the Transwell assay. Apoptosis in MKN45/DDP drug-resistant cells was detected using flow cytometry. The effect of a combination of EM and cisplatin on the levels of reactive oxygen species (ROS) and lipid peroxides (LPO) in cisplatin-resistant gastric cancer cells was detected using ROS fluorescent probes and a lipid peroxidation assay kit in conjunction with flow cytometry. The effect of EM combined with cisplatin on the level of iron ions was detected by fluorescence probe and confocal laser technique. Hematoxylin-eosin staining (HE staining) was used to detect the histopathologic morphology of drug-resistant gastric cancer in nude mice. Ferroptosis-related proteins were measured using immunohistochemistry. Real-time fluorescence quantitative polymerase chain reaction (RT-qPCR) was used to detect tumor drug resistance-related genes. The NF-κB/Snail pathway-related proteins, PI3K/AKT/mTOR pathway-related proteins, and drug resistance-related proteins were detected by Western blot.

Results and Conclusions: The results of in vitro and in vivo experiments showed that EM combined with DDP could effectively inhibit the migration and invasive ability of MKN45/DDP cells, as well as induce apoptosis of MKN45/DDP cells; the combination of the two drugs could significantly increase the levels of ROS, lipid peroxidation and divalent ferric ions in MKN45/DDP cells, at the same time reducing the levels of Ferroptosis-related proteins, which could induce Ferroptosis. In addition, EM combined with DDP can also exert the effect of reversing DDP resistance and increasing the sensitivity of gastric cancer drug-resistant cells to DDP by regulating the NF-κB/Snail signaling pathway, PI3K/AKT/mTOR signaling pathway, and the expression of drug resistance-related proteins and genes.

## INTRODUCTION

Gastric cancer (GC) is the fifth most common gastrointestinal malignancy and the fourth most common cause of death worldwide. Moreover, advanced gastric cancer has a high rate of metastasis and recurrence, and its 5-year survival rate is only about 25–30% [1], thus posing a great threat to human health. Due to the lack of obvious symptoms and specific markers for early diagnosis, most patients are diagnosed in the middle or late stages and miss the best time for surgery, so chemotherapy has become one of the most important treatments for gastric cancer. DDP is one of the most important chemotherapeutic drugs for patients with advanced gastric cancer and patients with metastatic cancer, and most patients can achieve better efficacy by DDP chemotherapy in the early stage, but the cytotoxicity of DDP on tumor cells decreases, and the effect of DDP on tumor cells decreases with the extension of the time of administration of DDP [2]. However, the cytotoxicity of DDP on tumor cells will be reduced with the prolongation of drug administration, which will lead to drug resistance [2], resulting in poor clinical therapeutic effects. Therefore, it is of great clinical significance and value to explore new drugs to reverse chemoresistance and sensitize and improve the efficacy of chemotherapy in gastric cancer to improve the quality of patients’ survival.

In recent years, traditional Chinese medicine (TCM) has been effective in improving drug resistance in tumor patients, alleviating the adverse effects of radiotherapy and chemotherapy, prolonging patient survival, etc. In particular, the combination of Chinese and Western medicines to inhibit tumor chemoresistance is the focus of current clinical oncology research in China [3, 4]. Li et al. [5] showed that paeonol inhibited the proliferation, migration, and glycolysis of lapatinib-resistant GC cells through LINC00665/miR-665/MAPK1 axis to inhibit the proliferation, migration, invasion, and glycolysis of lapatinib-resistant GC cells and promote apoptosis. *In vitro*, Xiao et al. [6] found that ginsenoside Rg3 sensitized GC cells to cisplatin treatment by upregulating miR-429 and inhibiting SOX2 and PI3K/AKT/mTOR signaling axes, especially when used in combination with cisplatin, which significantly increased the chemosensitivity of GC cells. Dihydroartemisinin (DHA) has anti-tumor resistance effects, Zhang et al. [7] indicated that DHA can enhance autophagy by inhibiting the PI3K/AKT/mTOR pathway, as well as induce apoptosis through caspase-dependent and mitochondrial pathways and by down-regulating P-glycoprotein (P-gp) to enhance cisplatin sensitivity.

Eremias multiocellata (EM) is a traditional Chinese herb that survives mainly in sandy areas and has the efficacy of activating blood circulation and removing blood stasis, eliminating gallstones and nodules, diuretic and diaphoretic, and sedative [8]. Some studies have shown that EM can play an anticancer role by inhibiting the expression of SIRT1 as well as the epithelial-mesenchymal transition-related proteins, such as E-cad and MMP9, in gastric cancer cells and inducing apoptosis [9, 10]. However, there are relatively few reports on the improvement of tumor chemoresistance by EM.

Therefore, in this study, the efficacy of EM combined with DDP in inhibiting the chemoresistance of gastric cancer was verified by combining *in vitro* and *in vivo* experiments, which will provide a reference for future research on its treatment of chemoresistance of gastric cancer.

## RESULTS

### Comparison of cell proliferative viability and drug resistance index between drug-resistant gastric cancer cell lines and parental lines

**Figure 1:**
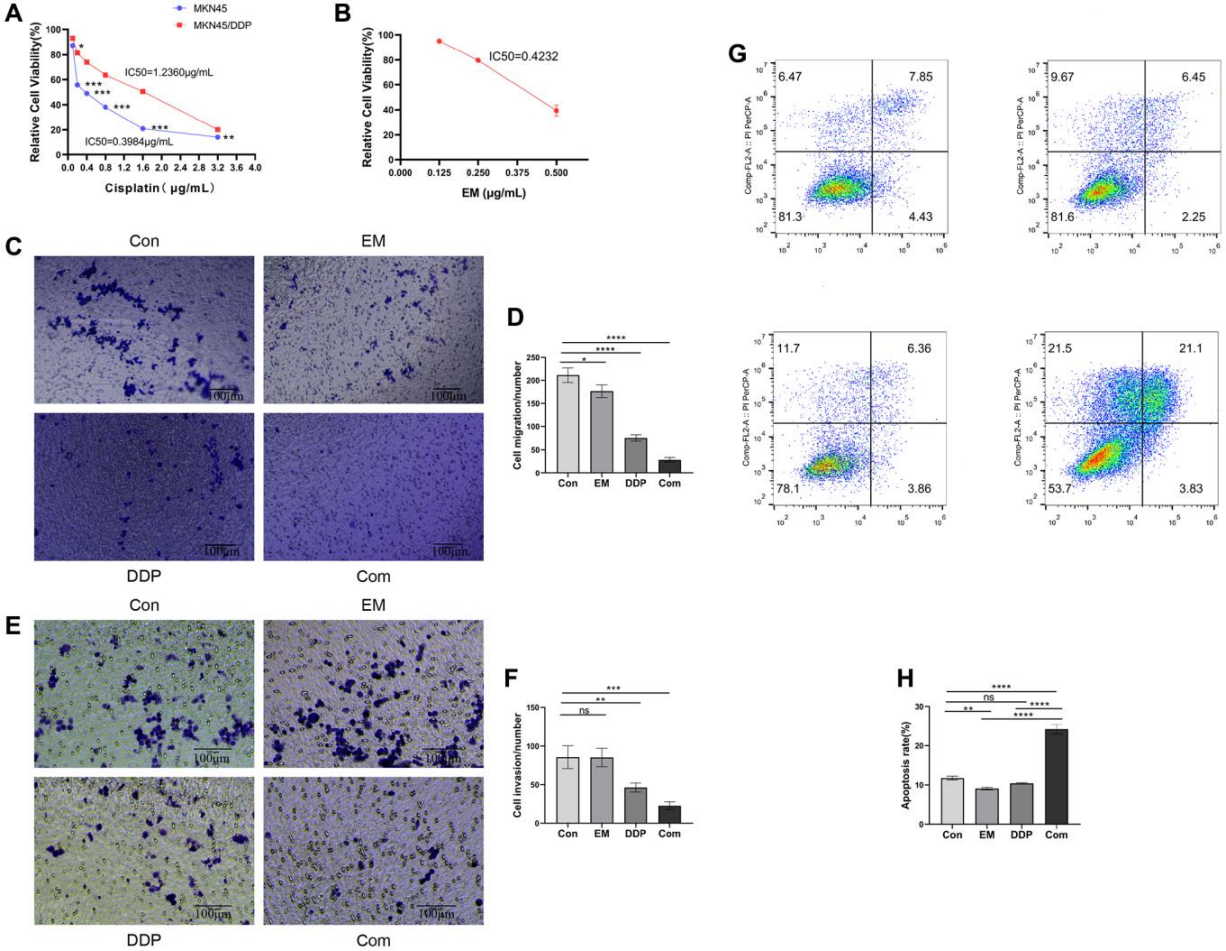
**Effect of EM combined with DDP on MKN45/DDP resistant cells.** (**A**) Impact of DDP on cell viability of MKN45 parental strain versus MKN45/DDP resistant strain. (**B**) Influence of EM on cell viability of MKN45/DDP resistant strain. (**C**, **D**) Affection of EM combined with DDP on the migration ability of MKN45/DDP cells. (**E**, **F**) Infection of EM combined with DDP on the invasion ability of MKN45/DDP cells. (**G**, **H**) Effect of EM combined with DDP on apoptosis of MKN45/DDP resistant cells. All experiments were reread three times, ^ns^*p* > 0.05, ^*^P < 0.05, ^**^P < 0.01, ^***^P < 0.001, ^****^P < 0.0001.

In order to determine the resistance of MKN45 and MKN45/DDP cells to DDP, we examined the proliferative viability of gastric cancer parental cell lines and drug-resistant cell lines at different concentrations of DDP (0.1, 0.2, 0.4, 0.8, 1.6, and 3.2 μg/mL) after 48 hours of effect by MTT assay. We found that DDP had a concentration-dependent inhibitory effect on MKN45 with an IC_50_ of 0.3984 μg/mL (Figure 1A); DDP exhibited an inhibitory effect on the proliferation of both MKN45/DDP cells with an IC_50_ of 1.2360 μg/mL (Figure 1A). Meanwhile, the proliferation viability of gastric cancer drug-resistant cell lines was higher than that of the parental strain regardless of the DDP concentration treatment (Figure 1A). The calculated resistance index (IC_50_ of MKN45/DDP cells)/(IC_50_ of MKN45-resistant cells) was 3.1024. Therefore, we demonstrated that the drug-resistant cell lines were less sensitive to DDP compared to the parental cell lines. The above results indicated that the drug-resistant gastric cancer cell lines had higher drug resistance while compared to the parental lines. In addition, a DDP of 1.2 μg/mL was selected for subsequent experiments within the range of pharmacological half-inhibitory concentrations considered.

### Effect of EM on the proliferation of MKN45/DDP Cells

We treated MKN45/DDP cells with EM at low concentration (0.125 μg/mL), medium concentration (0.25 μg/mL), and high concentration (0.5 μg/mL). After acting for 48 h, it was found that different concentrations of EM could inhibit the proliferation of gastric cancer drug-resistant cells, and there was a concentration dependence, the more obvious the inhibition of proliferation with the increase of drug concentration (Figure 1B). EM 0.25 μg/mL was selected for subsequent experiments within the range of pharmacological half-inhibitory concentrations considered.

### Effect of EM in combination with DDP on the migration and invasion ability of MKN45/DDP cells

To further investigate the effects of EM in combination with DDP on MKN45/DDP cells, we used the Transwell assay to measure the migration ability of MKN45/DDP cells. The results showed a significant decrease in the number of migrated cells in all treatment groups compared to the control group (Figure 1C, 1D, *P*-value: 0.0247, < 0.0001), with the combination group exhibiting the most pronounced inhibitory effect. Additionally, we assessed the invasion ability of MKN45/DDP cells treated with EM in combination with DDP, and the results demonstrated a significant reduction in the number of invaded cells in both the DDP group and the combination group compared to the control group (Figure 1E, 1F, *P*-value: 0.0065, 0.0003), with the combination group showing a greater reduction in invaded cells. These findings indicated that the combined treatment of EM and DDP effectively inhibited the migration and invasion ability of MKN45/DDP cells.

### Induction of apoptosis in MKN45/DDP resistant cells by EM in combination with DDP

Apoptosis analysis revealed a significant increase in the apoptosis rate of resistant cells in both the EM group and the combination group compared to the control group (Figure 1G, 1H, *P*-value: 0.0059, < 0.0001). Moreover, the combination group exhibited a significantly higher apoptosis rate of resistant cells compared to the EM group and the DDP group (Figure 1G, 1H, *P*-value: < 0.0001).

### Impact of EM combined with DDP on the levels of reactive oxygen species (ROS) and lipid peroxidation (LPO) in MKN45/DDP cells

**Figure 2:**
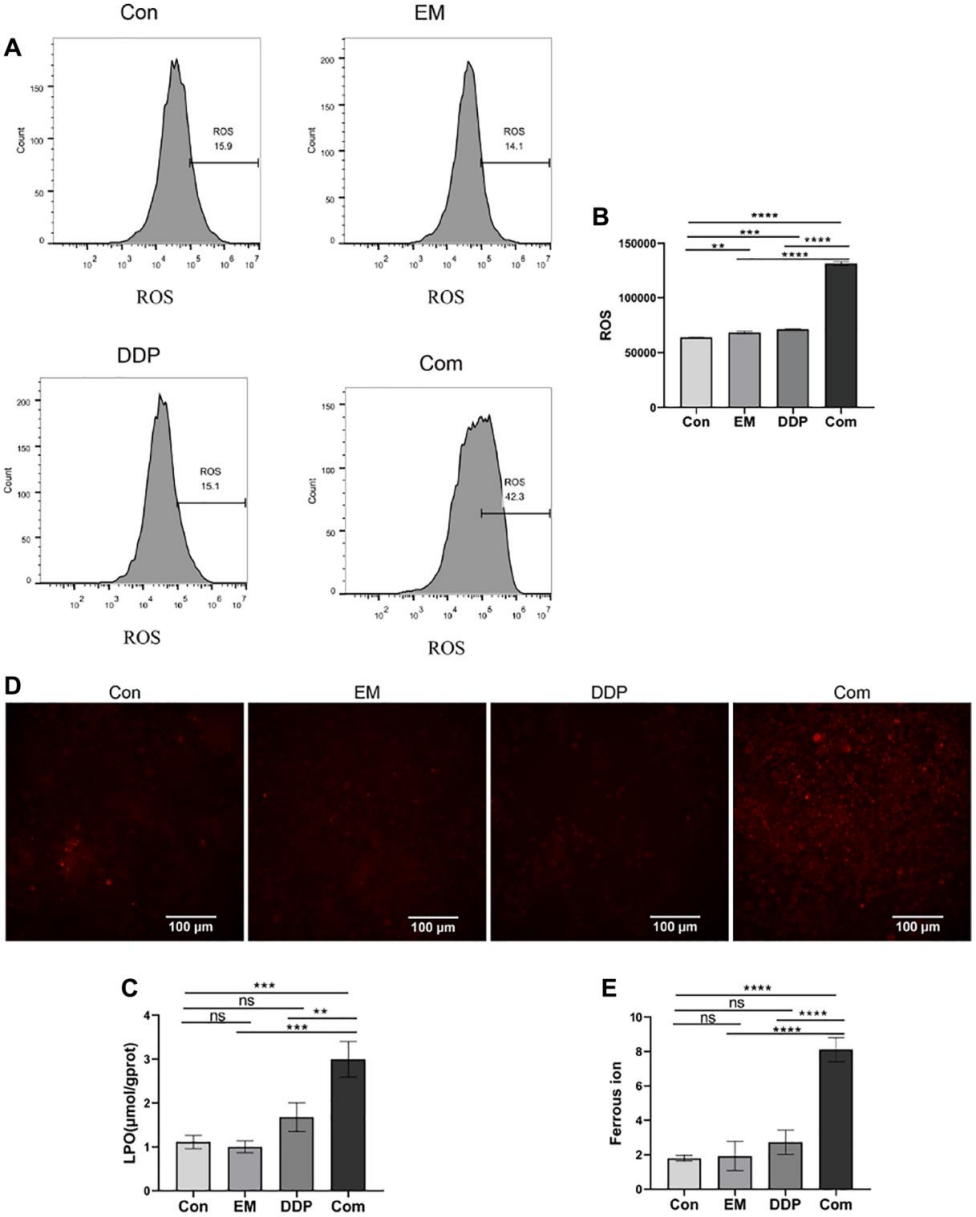
**The influence of EM combined with DDP on the indexes related to iron death.** (**A**) DCF fluorescence peak area plots of cells in each group. (**B**) Cells of each group ROS statistical results. (**C**) Results of LPO detection in cells of each group. (**D**) Fluorescence diagram of divalent iron ions in cells of each group. (**E**) Statistical results of the average fluorescence intensity of cells in each group. All experiments were reread three times, ^ns^*p* > 0.05, ^**^P < 0.01, ^***^P < 0.001, ^****^P < 0.0001.

To clarify the effects of EM, DDP, and the combination of the two drugs on the level of ROS in MKN45/DDP cells, we detected ROS in the cells using a fluorescent probe, DCFH-DA. The DCFH-DA probe is non-fluorescent by itself and has the ability to freely pass through the cell membrane and enter the cell, which is confined to the cell when it is hydrolyzed by the cellular cytosol esterase to become DCFH. Subsequent oxidation by ROS generates fluorescent DCF. Based on the above properties of the DCFH-DA probe, the intracellular ROS level can be detected in combination with flow cytometry. According to the previously described experimental method, we examined the ROS levels of cells in different treatment groups, and the results are shown in Figure 2A, 2B. We can see that the intracellular ROS levels were significantly higher in the EM, DDP, and combined group compared with the control group (*P*-value: 0.0050, 0.0001, < 0.0001), and the combined group had a significantly higher ROS level than that of the EM and DDP groups (*P*-value: < 0.0001). The above results showed that the ROS level of cisplatin-resistant gastric cancer cells could be significantly improved under the effect of 0.25 μg/mL EM combined with 1.2 μg/mL DDP.

The increased level of intracellular LPO is one of the characteristic events of Ferroptosis. Therefore, we examined the LPO levels in the above cell groups, and the results are shown in Figure 2C. From the results, we found that compared with the control group, the intracellular LPO levels in the combined group were all significantly higher (*P*-value: 0.0002), and the LPO levels in the combined group were higher than those in the EM and DDP groups (*P*-value: 0.0001, 0.0019). The above results demonstrated that the intracellular LPO levels of MKN45/DDP cisplatin-resistant gastric cancer cells were obviously increased by the combination of 0.25 μg/mL EM and 1.2 μg/mL DDP.

### The role of EM combined with DDP on divalent iron ion levels in MKN45/DDP cells

We examined the divalent iron ion levels in the cells of control, EM, and DDP, as well as combined groups using laser confocal and RhoNox-1 fluorescent probes, and the results are shown in Figure 2D, 2E. From the figures, we found that the fluorescence intensity in the cells of the combined group was greatly enhanced compared with that of the control group (*P*-value: < 0.0001), and the fluorescence intensity of the combined group was clearly higher than that of the EM group and the DDP group (*P*-value: < 0.0001). This result demonstrated that EM combined with DDP could promote the accumulation of divalent iron ions in gastric cancer cisplatin-resistant cells MKN45/DDP to a certain extent.

### Inhibitory effect of EM combined with DDP on the cisplatin-resistant subcutaneous tumor of gastric cancer in nude mice

**Figure 3:**
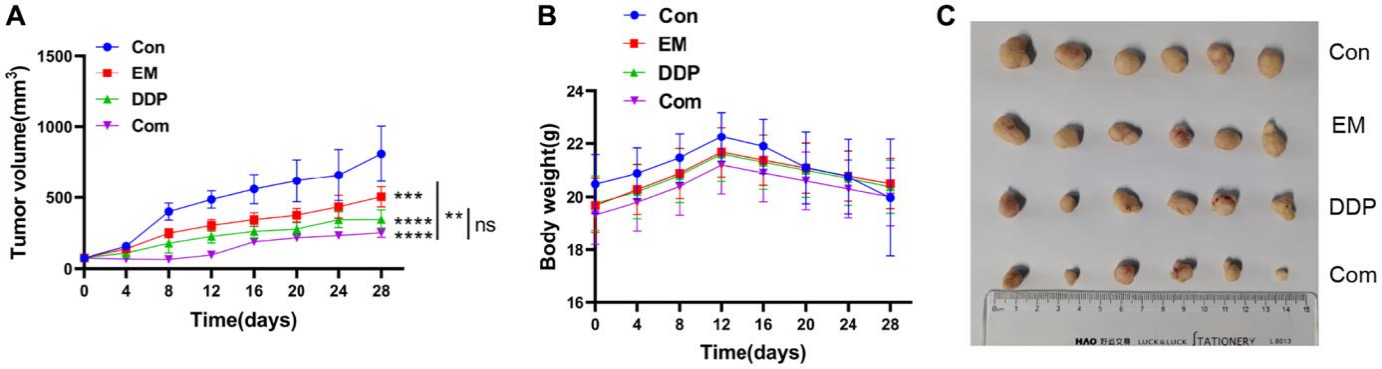
**Inhibitory effect of EM combined with DDP on the cisplatin-resistant subcutaneous tumor of gastric cancer in nude mice.** (**A**) Tumor growth graph of nude mice in each group. (**B**) Line graph of body weight of nude mice in each group. (**C**) Tumor size of nude mice in each group. ^**^P < 0.01, ^***^P < 0.001, ^****^P < 0.0001.

To validate the inhibitory influence of the combination of EM and DDP on cisplatin-resistant gastric cancer subcutaneous tumors in nude mice, we measured the body weight and tumor volume of each group of mice every 3 days after drug administration and plotted the tumor volume growth curve. The results (Figure 3A) showed that compared to the control group, the EM group, DDP group, and combination group all exhibited significant inhibition of tumor growth (*P*-value: 0.0006, < 0.0001). Notably, the combination group showed a slower tumor growth rate compared to the EM group (*P*-value: 0.0039). Safety evaluation of the drug combination was also assessed by monitoring the changes in body weight of the mice in each group. The results (Figure 3B) demonstrated that after completion of the treatment, there were no obvious differences in body weight between the EM group, DDP group, combination group, and control group (*P* > 0.05). These findings indicated that both individual administration and combination treatment with DDP at 2 μg/kg and EM at 2 g/kg exerted an apparent inhibitory effect on cisplatin-resistant gastric cancer subcutaneous tumors in nude mice without causing noticeable toxicity.

Furthermore, we administered the drug at the end of the treatment complete peeling of the nude mice in each group of tumor tissue (Figure 3C), it can be seen that it is round or round-like, brittle, hard, clear boundaries, and the surface of some of the tumor tissues are easy to break and bleed; measurement of the volume of the tumor in each group: it was found that the volume of the tumor was larger in the control group, but the volume of the tumor in the DDP group and the combined group were smaller, among which the volume of the tumor in the combined group was the smallest, which indicated that the treatment influence of EM combined with the DDP group was better.

### Impact of EM and DDP on the histopathological changes of tumors in drug-resistant nude mice with gastric cancer

**Figure 4:**
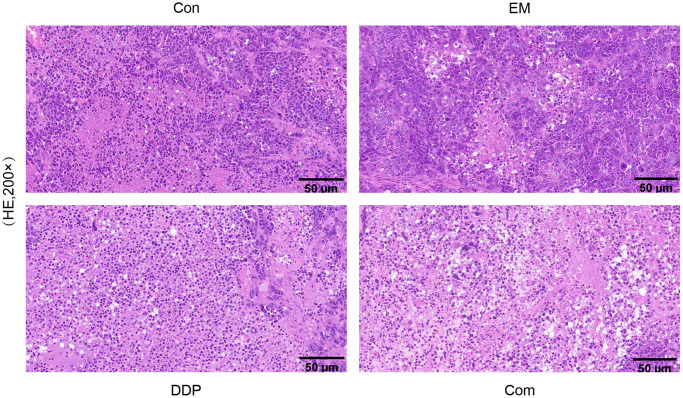
The histopathological morphological changes in tumor tissues of each group of nude mice.

The results of HE staining showed that the tumor tissues in the model group were round, ovoid or irregular, and closely connected; the nuclei of the cells were large, deeply stained, rounded, and uniform, while the tumor cells in the EM group, the DDP group as well as the combination group were more sparsely arranged, with different sizes, and showed flaky necrosis, and the nuclei of the cells appeared to have different degrees of shrinkage or breakage. The above results indicated that EM, DDP, and the combination of two drugs could promote apoptosis of gastric cancer drug-resistant cells to different degrees (Figure 4).

### The combination of EM and DDP can reduce the protein levels of GPX4 and FTH1 in nude mice with cisplatin-resistant gastric cancer

**Figure 5:**
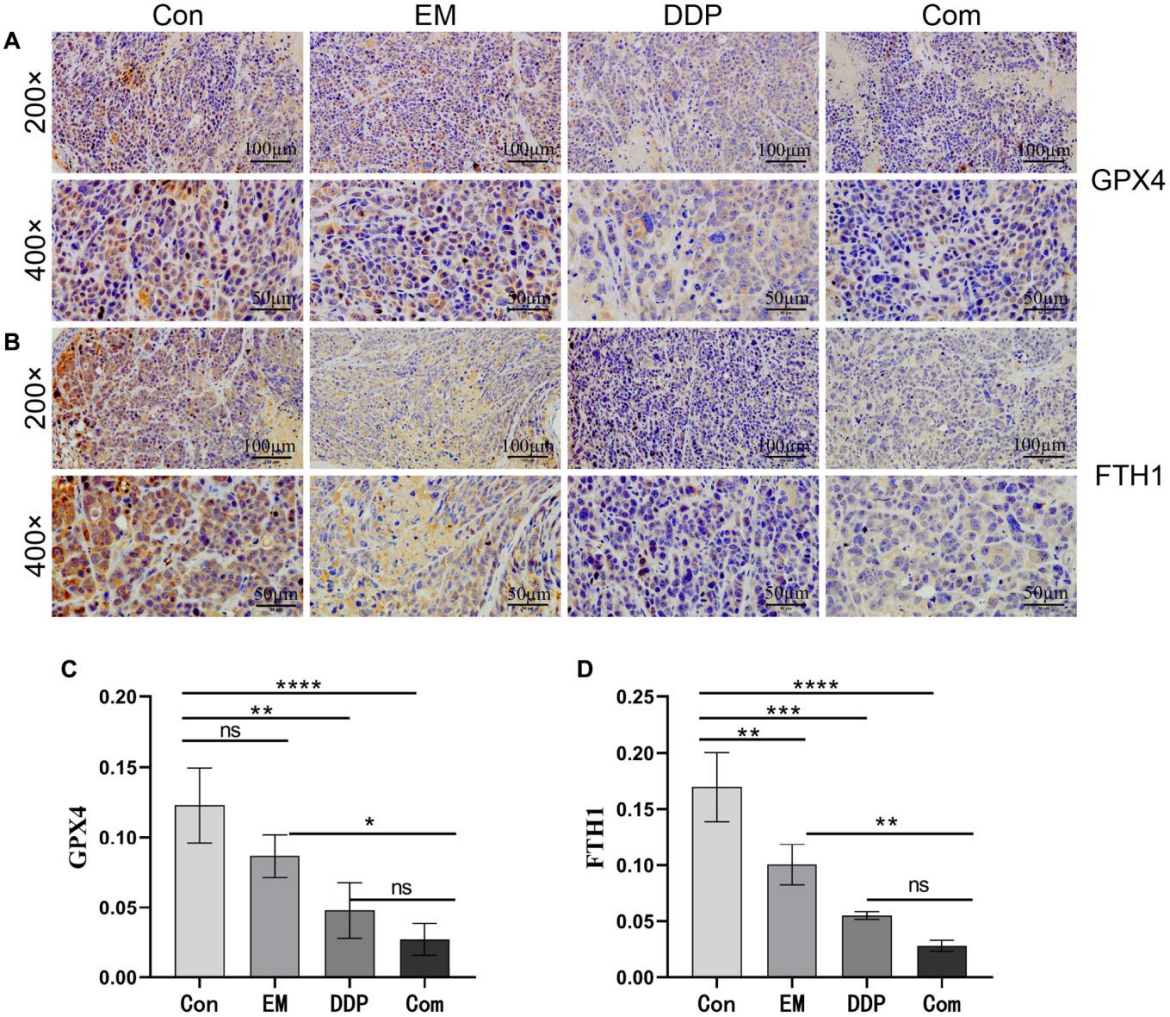
**Effect of EM combined with DDP on GPX4 and FTH1 protein levels.** (**A**, **B**) GPX4 and FTH1 protein expression in tumor tissues of nude mice in each group. (**C**, **D**) Graphs of GPX4 and FTH1 protein expression. All experiments were reread three times, ^ns^*p* > 0.05, ^*^P < 0.05, ^**^P < 0.01, ^***^P < 0.001, ^****^P < 0.0001.

GPX4 and FTH1 are the symbols of iron death occurring in tumor cells. Therefore, we used immunohistochemistry to detect the effect of EM combined with DDP on the protein expression of GPX4 and FTH1. The positive expression of GPX4 and FTH1 was mostly concentrated in the cytoplasm or cell membrane, with brownish-yellow or brown granular color, and was mostly diffusely distributed in sheets in the field of view of the tissue. The results showed that (Figure 5A–5D) compared with the control group, the positive expression of GPX4 (*P*-value: 0.0059) and FTH1 (*P*-value: 0.0069, 0.0002) proteins in the EM group, the DDP group, and the combined group were reduced sequentially; At the same time, GPX4 (*P*-value: 0.0218) and FTH1 (*P*-value: 0.0053) in the combined group were lower than those in the EM group. The above results concluded that EM combined with DDP could play a role in inhibiting cisplatin resistance in gastric cancer nude mice by decreasing the expression of GPX4 and FTH1 proteins, thereby inducing cells to produce iron death.

### The combination of EM and DDP can reduce the expression of tumor drug resistance-related genes P-gp and MRP6

**Figure 6:**
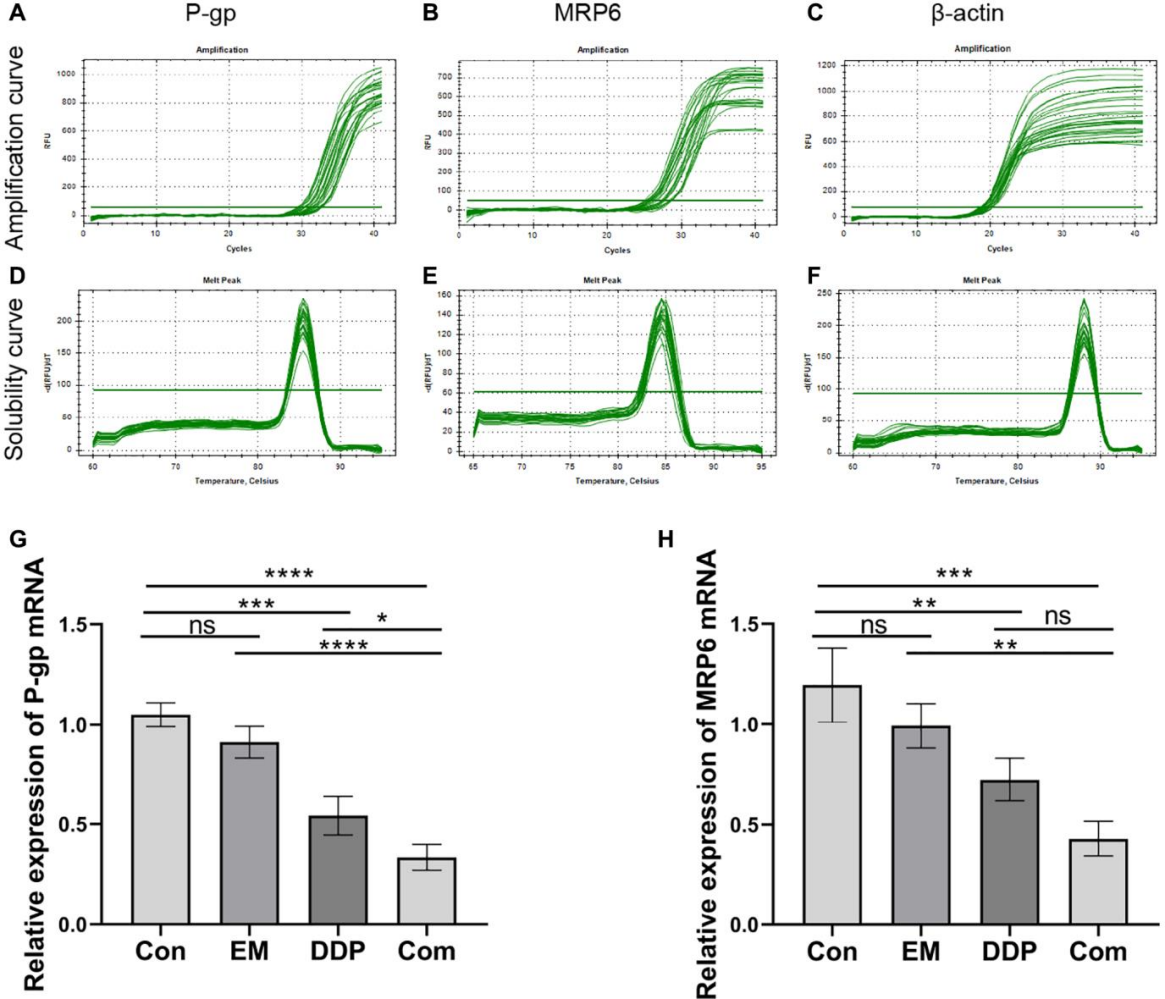
**The mRNA expression of P-gp and MRP6 by EM combined with DDP.** (**A**–**C**) Amplification curve of P-gp, MRP6, and β-actin. (**D**–**F**) Solubility curve of P-gp, MRP6, and β-actin. (**G**) Relative mRNA expression of P-gp. (**H**) Relative mRNA expression of MRP6. All experiments were repeated three times, ^ns^*p* > 0.05, ^*^P < 0.05, ^**^P < 0.01, ^***^P < 0.001, ^****^P < 0.0001.

P-gp and MRP6 are an important class of transmembrane transporter genes that play a key role in tumor cells and can regulate the resistance of tumor cells to chemotherapeutic drugs. We used RT-qPCR to detect the effect of EM combined with DDP on the expression of tumor drug resistance-related genes P-gp and MRP6 at the gene level, also drawn the amplification and solubility curve (Figure 6A–6F). The results showed (Figure 6G, 6H) that the mRNA expression of P-gp (*P*-value: 0.0002, < 0.0001) and MRP6 (*P*-value: 0.0086, 0.0004) were significantly reduced in the DDP and combination groups compared with the control group; meanwhile, the combination group had significantly lower mRNA expression of P-gp (*P*-value: < 0.0001, 0.0408) and MRP6 (*P*-value: 0.0029) than those in the EM and DDP groups. The above results indicated that EM combined with DDP could inhibit the expression of P-gp and MRP6 at the gene level to different degrees, and thus inhibit cisplatin resistance in gastric cancer.

### EM combined with DDP improved chemoresistance in gastric cancer nude mice by modulating the NF-κB/Snail pathway, the expression of resistance-associated proteins, and the PI3K/AKT/mTOR pathway

**Figure 7:**
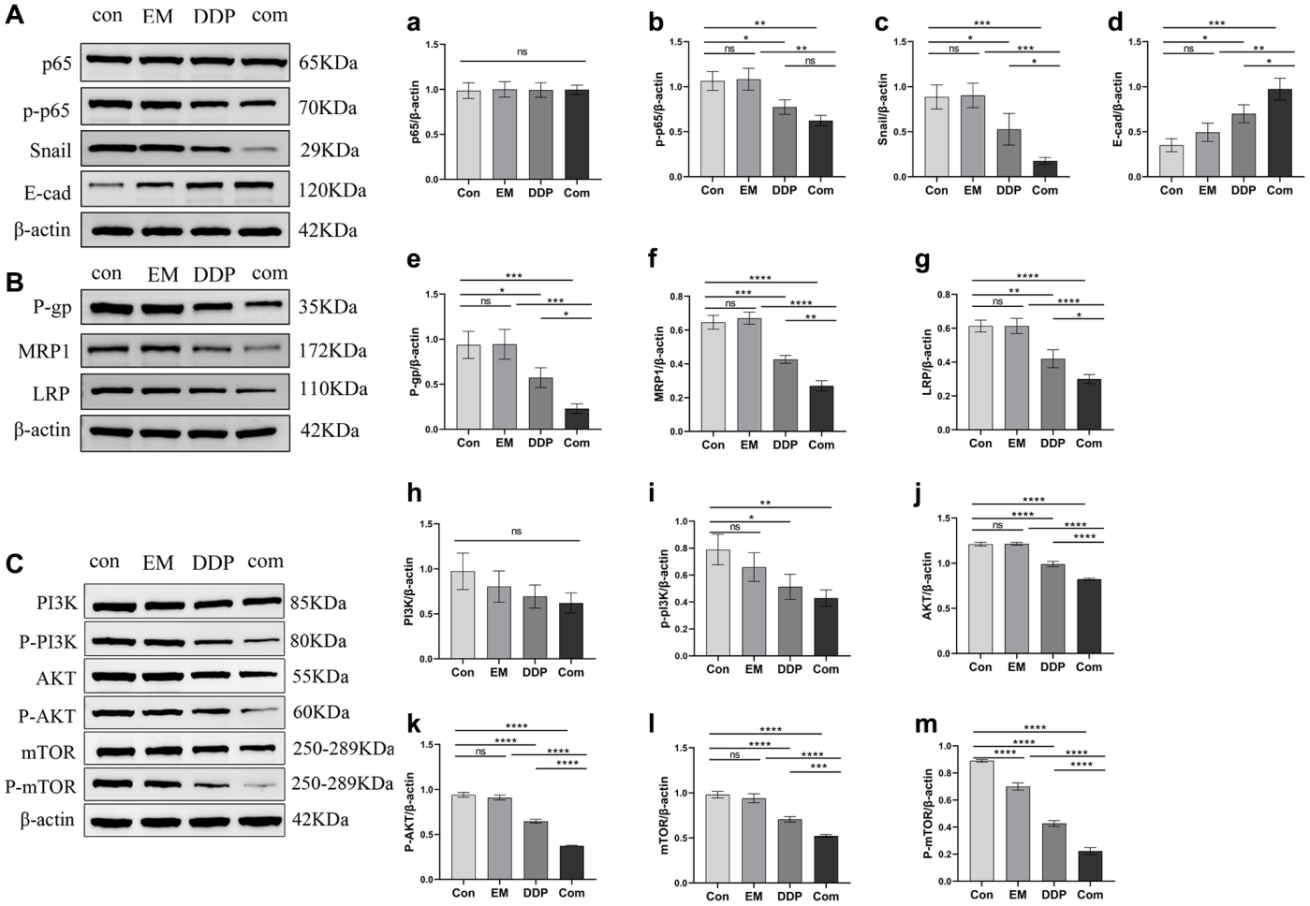
**Effect of EM combined with DDP on the NF-κB/Snail pathway and expression of drug resistance-related proteins.** (**A**, **B**): Western plots of P65, p-p65, Snail, E-cad, P-gp, MRP1, LRP proteins. (**a**) Expression of p65 protein. (**b**) Expression of p-p65 protein. (**c**) Expression of Snail protein. (**d**) Expression of E-cad protein. (**e**) Expression of P-gp protein. (**f**) Expression of MRP1 protein. (**g**) Expression of LRP protein. (**C**): Western plots of PI3K, AKT, mTOR, P-PI3K, P-AKT, and P-mTOR proteins. (**h**) Expression of PI3K protein. (**i**) Expression of P-PI3K protein. (**j**) Expression of AKT protein. (**k**) Expression of P-AKT protein. (**l**) Expression of mTOR protein. (**m**) Expression of P-mTOR protein. All experiments were repeated three times. ^ns^*p* > 0.05, ^*^P < 0.05, ^**^P < 0.01, ^***^P < 0.001, ^****^P < 0.0001.

The NF-κB/Snail signaling pathway and the drug resistance-associated proteins P-gp, MRP1, and LRP play important roles in tumor drug resistance. We used Western blotting to detect changes in the expression of p65, phosphorylated p65 (p-p65), Snail, E-cad, and drug resistance-associated proteins P-gp, MRP1, and LRP in tumor tissues (Figure 7A, 7B, a–g). The results showed that compared with the control group, the EM combined with the DDP group were able to differentially down-regulate the protein expression levels of p-p65 (*P*-value: 0.0020), Snail (*P*-value: 0.0007), P-gp (*P*-value: 0.0006), MRP1 (*P*-value: < 0.0001) and LRP (*P*-value: < 0.0001), as well as significantly up-regulate the protein expression level of E-cad (*P*-value: 0.0003). Meanwhile, the expression levels of p-p65 (*P*-value: 0.0015), Snail (*P*-value: 0.0006, 0.0447), P-gp (*P*-value: 0.0006, 0.0432), MRP1 (*P*-value: < 0.0001, 0.0018) and LRP (*P*-value: < 0.0001, 0.0298) proteins in the EM combined DDP group were significantly lower than those in the EM and DDP groups, and the expression level of E-cad (*P*-value: 0.0016, 0.0402) protein was higher than in the EM and DDP groups. However, EM combined with DDP had no significant effect on the total P65 protein (*P >* 0.05).

Moreover, the PI3K/AKT/mTOR pathway also has a critical position in tumor drug resistance. Our experimental results showed (Figure 7C, h–m) that both EM combined with DDP groups were able to significantly downregulate the levels of p-PI3K (*P*-value: 0.0074), AKT (*P*-value: < 0.0001), p-AKT (*P*-value: *P*-value: < 0.0001), mTOR (*P*-value: < 0.0001), and p-mTOR (*P*-value: < 0.0001) protein expression compared with the control group; The Com group was able to further downregulate AKT (*P*-value: < 0.0001), p-AKT (*P*-value: < 0.0001), mTOR (*P*-value: < 0.0001, 0.0010), p-mTOR (*P*-value: < 0.0001) protein expression levels compared to the EM and DDP monotherapy groups. EM combined with DDP slightly downregulated the total protein expression level of PI3K; But the difference was not statistically significant (*P* > 0.05).

## DISCUSSION

GC is a malignant lesion of the gastric mucosa caused by Helicobacter pylori infection (HP infection), diet, genetics, environment, and other factors. Its morbidity and mortality rates are high in the world and have a tendency to increase year by year. Most of the clinical treatments for GC use DDP as the basic chemotherapeutic drug while combined with other types of drugs, but with the prolongation of the time of drug use, GC cells become resistant to DDP, thus reducing the clinical efficacy to a large extent. In recent years, DDP has been used in combination with other modulators to treat many tumors by targeting apoptosis or other cancer signaling pathways. Therefore, the present study was designed to investigate the effect of EM combined with DDP on GC-resistant cells based on previous work and to tentatively hypothesize its mechanism of action.

DDP is a cytotoxic, cell cycle non-specific drug widely used in pre-operative chemotherapy, palliative care, late-stage treatment, and metastatic tumor treatment, which works by inhibiting the replication of DNA of tumor cells and damaging their cell membrane structure, thereby exhibiting strong broad-spectrum anticancer effects [11]. EM is a traditional Chinese herbal medicine that also serves as an animal medicine. Its functions include activating blood circulation and relieving blood congestion, removing gallstones and dispersing nodules, and having diuretic and sedative effects [8]. This study aims to examine the efficacy of the combined use of DDP and EM in treating GC chemoresistance through a series of *in vitro* and *in vivo* experiments to provide some clues for future studies on the treatment of GC chemoresistance.

In order to elucidate the resistance of MKN45 cells and MKN45/DDP cells to DDP, we used the MTT assay to determine the proliferative viability of the parental and resistant GC cell lines after 48 h of exposure to DDP at different concentrations. We found that DDP had a concentration-dependent inhibitory effect on MKN45 cells with an IC_50_ of 0.3984 μg/mL. Meanwhile, DDP also showed an inhibitory effect on the proliferation of MKN45/DDP cells with an IC_50_ of 1.2360 μg/mL. In addition, the proliferative viability of the GC-resistant cell lines was higher than that of the parental cell lines regardless of the concentration of DDP. The resistance index was calculated to be 3.1024. The above results indicated that the GC-resistant cell line has higher resistance and reduced sensitivity to DDP compared to the parental cell line. Additionally, in order to demonstrate the impact of EM on the proliferative viability of MKN45/DDP cells, we treated them with varying concentrations of EM. After 48 hours, it was found that different concentrations of EM could suppress the proliferation of GC-resistant cells and that this effect was concentration dependent, with more pronounced suppression observed at higher concentrations of the drug. However, taking into account the pharmacological half-inhibitory concentration range, doses of 1.2 μg/mL for DDP and 0.25 μg/mL for EM were chosen for the subsequent experiments.

To further elucidate the mechanism of action of EM combined with DDP for the treatment of chemoresistance in gastric cancer, we used the Transwell method to detect cell migration and invasion ability. The results showed that compared with the control group, the number of migrating and invading cells was reduced to different degrees in each treatment group, among which the reduction was more obvious in the combination group. The above results indicated that EM combined with DDP could effectively inhibit the migration and invasion ability of MKN45/DDP cells. Apoptosis is involved in tumor development as a death mechanism regulated by multiple apoptotic genes [12]. Our *in vitro* experiments showed that the apoptosis rate of drug-resistant cells increased to varying degrees in each treatment group compared to the control group, with the highest rate observed in the combination group. At the same time, we also performed HE staining for *in vivo* experiments, and the results showed that compared with the control group, the tumor cells in the EM group, the DDP group, and the combination group were more sparsely arranged, varied in size, showed sheet necrosis, and the nuclei appeared to be shrunk or broken to different degrees. All of the above results indicated that EM, DDP, and the combination of the two drugs could significantly induce apoptosis of MKN45/DDP cells. What’s more, to verify that EM combined with DDP could effectively inhibit the growth of subcutaneous tumors in cisplatin-resistant nude mice with gastric cancer, we measured the body weights and tumor sizes of the nude mice every 3 days and plotted the tumor volume growth curves. The results showed that compared with the control group, the EM group, the DDP group, and the combination group could inhibit the growth rate of the tumors, with the combination group having the slowest tumor growth rate. While determining the efficacy of dense point EM combined with DDP treatment, we also evaluated the safety of the combination of the two drugs by the change in body weight of nude mice. The results showed that there was no significant difference in the body weights of nude mice in the EM, DDP, and combination group compared to the control group. In conclusion, all the above results showed that DDP 2 μg/kg and EM 2 g/kg alone and in combination were safe and effective with no significant toxic effects.

The NF-κB/Snail signaling pathway is closely related to tumor development. NF-κB is an important nuclear transcription factor that can be involved in the inflammatory response and immune response of the organism and can regulate apoptosis and stress response. At the same time, the overactivation of NF-κB has been associated with many human diseases, such as tumors and rheumatoid arthritis, etc., [13, 14]. Therefore, inhibition of the NF-κB pathway has become an important approach for the treatment of related diseases. NF-κB, a promoter of tumourigenesis and development, has been associated with multidrug resistance (MDR) in tumors. The study identified a shared NF-κB binding site in the first intron of the MDR1 gene and demonstrated that complexes of NF-κB can bind to this intronic site to initiate the transcription of MDR1, leading to the development of drug resistance [15]. In addition, NF-κB is mainly composed of nuclear transcription factors p65 and p50 as homo-/heterodimers, which can coordinate a variety of gene expressions and influence biological functions such as cell differentiation, apoptosis, and tumor growth *in vivo*, with phosphorylation of p65 being one of the most critical modifications of NF-κB [16–18]. What’s more, NF-κB has been shown to regulate epithelial-mesenchymal transition (EMT) in tumor cells. It promoted the metastasis of tumor cells by inducing the activation of EMT, and after EMT was induced, the level of E-cadherin was significantly reduced, while the levels of N-cadherin and poikilodulin are increased [19]. Snail is a member of the Snail superfamily of zinc finger transcriptional regulators that induce EMT, down-regulate E-cadherin expression, and are involved in tumor infiltration and metastasis, and plays an important role in regulating tumor development and metastasis [20, 21]. It has been shown [22] that Snail is highly expressed in gastric cancer tissue and is closely associated with histological grading and tumor drug resistance. Our experimental results showed that p-p65 and Snail proteins were highly expressed and E-cadherin protein was lowly expressed after cisplatin resistance in gastric cancer, and after EM combined with DDP, the expressions of p-p65 and Snail were downregulated to different degrees, and the expression of E-cadherin was upregulated to different degrees. The above results indicated that EM combined with DDP could inhibit the EMT process and further increase the sensitivity of gastric cancer-resistant cells to cisplatin by mediating the NF-κB/Snail signaling pathway to different degrees, which led to the downregulation of p-p65 and Snail expression and the upregulation of E-cadherin, thus improving the sensitivity of gastric cancer-resistant cells to cisplatin.

Furthermore, the PI3K/AKT/mTOR signaling pathway is a fundamental pathway in cancer development, and its abnormal activation significantly contributes to the chemoresistance exhibited by tumor cells. The lipid kinase PI3K can induce the activation of its downstream pathway AKT under certain conditions, and the activated AKT can initiate signaling pathways, regulate downstream target genes, and generate a cascade of responses, thereby regulating tumor cell proliferation, differentiation, metabolism, and migration, which promoted drug resistance in tumor cells [23, 24]. mTOR plays a crucial role as a regulatory molecule in triggering autophagy and is a component of the downstream channel of this pathway. As a member of the PI3K-related kinase protein family, AKT can stimulate cell growth while restraining apoptosis and autophagy through the activation of mTOR phosphorylation [25]. Our study found that the proteins p-PI3K, p-AKT, and p-mTOR were highly expressed in DDP-resistant gastric cancer. However, their expression was significantly reduced after EM and DDP treatment. The aforementioned findings indicated that EM in combination with DDP ameliorated the chemoresistance of DDP in gastric cancer by regulating the PI3K/AKT/mTOR signaling pathway, thus the combination of the two drugs acts as a sensitizer and enhancer.

Multiple drug resistance (MDR) in tumor cells is often associated with the overexpression of ATP-binding cassette (ABC) transporter proteins, including P-glycoprotein (P-gp), multidrug resistance-associated protein 1 (MRP1), multidrug resistance-associated protein 6 (MRP6) and lung resistance-associated protein (LRP), which mediate tumor cell resistance to chemotherapeutic drugs and inhibit tumor cell killing by reducing the concentration of chemotherapeutic drugs in the cells [26–30]. Our experimental results showed that P-gp, MRP1, MRP6 and LRP showed high expression after cisplatin resistance in gastric cancer, but the expression of the above proteins and genes showed different degrees of down-regulation after the administration of EM combined with DDP. This indicated that EM combined with DDP could inhibit the expression of exocytosis proteins and genes to different degrees and improve the chemotherapy resistance of gastric cancer.

In recent years, Ferroptosis has been identified as a novel modality of regulated cell death based on the accumulation of intracellular iron ions (mainly divalent iron ions) and lipid peroxidation (LPO) [31]. It has been shown that iron ions aggregated during Ferroptosis can generate large amounts of reactive oxygen radicals (ROS) via the Fenton reaction, triggering the accumulation of lipid peroxides and ultimately leading to cell death [32, 33]. Meanwhile, Ferroptosis has been reported to be closely associated with the accumulation of ROS and the reversal of cisplatin resistance [34]. All of the above studies suggest that ROS is a key factor in cellular iron death. In addition, glutathione peroxidase 4 (GPX4) and ferritin heavy chain 1 (FTH1) are key proteins in the development of iron death and are important regulators of Ferroptosis [35]. Chen et al. [36] found that curcumin analog-induced androgen receptor ubiquitination induced Ferroptosis and ameliorated glioblastoma resistance to temozolomide via downregulation of GPX4. Wang et al. [37] found that the Wnt/conjugated protein signaling pathway controls iron pituitary disease by targeting and regulating the level of GPX4, which in turn promotes chemotherapeutic resistance in tumor cells. Our experimental results showed that EM combined with DDP significantly increased the levels of LPO, ROS, and divalent iron ions in MKN45/DDP cells while decreasing the levels of GPX4 and FTH1 proteins in the subcutaneous tumor-resistant nude mouse model of gastric cancer. Therefore, we concluded that the combination of the two drugs may have a role in the reversal of cisplatin resistance in gastric cancer through the induction of cellular Ferroptosis.

In summary, we initially clarified the mechanism of EM combined with DDP to overcome cisplatin resistance in gastric cancer, which may be that the combination of the two drugs inhibited the migration and invasion ability of drug-resistant cells, promoted apoptosis of drug-resistant cells, and down-regulated the expression of drug resistance-related proteins and genes through the NF-κB/Snail signaling pathway, thus inhibiting the proliferation of drug-resistant cells in gastric cancer. What’s more, EM combined with DDP may promote the accumulation of intracellular divalent iron ions by inducing the downregulation of the expression of GPX4 and FTH1, resulting in the overaccumulation of intracellular ROS and LPO levels, and ultimately inducing Ferroptosis in MKN45/DDP cells, which in turn overcomes cisplatin resistance in gastric cancer. However, the extraction and compositional identification of EM is still a challenge due to the fact that there are fewer studies on the related drugs, the control that can be used for mass spectrometry has not yet been inquired, and its specific composition is still not clear. Therefore, our group is still working on EM extraction and compositional identification.

## MATERIALS AND METHODS

### Laboratory animals and materials

SPF-grade BALB/c nude mice (male, 4–6 weeks old), body mass (20 ± 2) g, were purchased from Beijing Viton Lever Laboratory Animal Technology Company, China. All nude mice were housed in the Animal Centre of Ningxia Medical University, where the controlled temperature was maintained at (22 ± 3)°C and relative humidity at (50 ± 10)% during a 24-h light/dark cycle, the diet was provided by the laboratory, and experiments were performed after 1 week of acclimatization feeding. The animal experiments were approved by the Ethical Review Committee of Ningxia Medical University (No. IACUC-NYLAC-2022-109). MKN45 cells and MKN45/DDP were purchased from Zhejiang Meisen Cell Technology Company, China. MTT cell proliferation and cytotoxicity assay kits were purchased from Wuhan Boster Bioengineering Company (CAT: KGA311-KGA312, Wuhan, China). RPMI 1640 medium and penicillin-streptomycin mixture were purchased from Shanghai Tercel Biotechnology Company (CAT: 2230182, 20220705, Shanghai, China). Fetal bovine serum was purchased from Beijing Solarbio Technology Company (CAT: 2222092, Beijing, China). Transwell nesting was purchased from Corning (CAT: 3422, Shanghai, China). An apoptosis detection kit was purchased from Wuhan Servicebio (CAT: G1511, Hubei, China). DCFH-DA probe was purchased from Shang Biotechnology (CAT: S0033, Shanghai, China). RhoNox-1 working solution was purchased from Shanghai MCE Biotechnology (CAT: HY-D1533 Shanghai, China). A lipid peroxidation assay kit was purchased from Nanjing Jianjian Bioengineering Institute (CAT: A106-1, Jiangsu, China). Hematoxylin-eosin (HE) staining solution was purchased from Wuhan Servicebio (CAT: CR2107043, Hubei, China). P65, P-P65, P-gP, LRP antibodies were purchased from Wuhan Servicebio (CAT: GB11997, GB113882, GB113291, GB112334, Hubei, China); Snail, E-Cad, GPX4, FTH1 antibodies were purchased from Jiangsu Affinity Biotechnology Company (CAT: AF6032, AF0131, DF6701, DF6278, Jiangsu, China). MRP1 was purchased from Shenzhen Genetex Biotechnology Company (CAT: GTX116046, Guangdong, China). PI3K, p-PI3K, AKT, p-AKT, mTOR, and p-mTOR antibodies were purchased from Jiangsu Affinity Biotechnology Company (CAT: AF6241, AF3241, AF6261, AF0016, AF6308, AF3308, Jiangsu, China). The immunohistochemistry kit was purchased from Beijing Zhongsui Jinqiao Biotechnology Company (CAT: 2206C0809, Beijing, China).

### Preparation of EM extract

EM was obtained from the Hospital of Traditional Chinese Medicine, Ningxia Medical University. Cell experiments: 100 g of EM was weighed and crushed into a fine powder, added with ultrapure water, and fixed to 1000 mL, its material-liquid ratio was 1:10, and extracted for 4 h with a 4°C low-temperature ultrasonic crusher. The extracted mixture was centrifuged at 5500 rpm, 4°C, for 30 min, and then the precipitate was taken to be alcohol-sedimented in 55% ethanol at 4°C for 4 h. The supernatant was taken after centrifugation as above. The mixture was spun back and evaporated to be alcohol-free at 37°C, and then dried in a vacuum freeze dryer at −80°C for 24 h into powder form. Next, the solution was configured with RPMI 1640 medium at a concentration of 1 mg·mL-1 and frozen at −80°C. Animal experiment: The traditional method of decoction was used, EM was added to distilled water and fixed at 1000 mL, then immersed for 40 minutes. It was then placed in a saucepan at 100°C and boiled, then reduced to 80°C. When the decoction reached 500 mL, it was filtered through gauze, 800 mL of distilled water was added for the second decoction, and finally the two decoctions were combined. Based on the results of the previous pre-tests, the EM was concentrated to an optimal raw drug concentration of 0.026 g·mL-1 in aqueous solution, which was bottled and stored at 4°C for reserve use.

### Preparation of DDP solution

Cellular assay: DDP powder was diluted to 1 μg·uL^−1^ with PBS. Animal assay: DDP powder was diluted to 0.2 mg·mL^−1^ with saline.

### Cell culture

MKN45/DDP cells were cultured in RPMI 1640 complete medium containing 5 μmol/L DDP to maintain their drug resistance. They were cultured in a cell culture incubator at 37°C with 5% CO_2_. The cells were switched to DDP-free complete medium 2 weeks before the experiment, and the cells in the logarithmic growth phase were used for the experiment.

### MTT experiment

Cells of MKN45 and MKN45/DDP were inoculated into 96-well plates at a concentration of 1 × 10^4^ cells/ml during logarithmic growth. These were cultured in a cell culture incubator for 24 hours. Afterward, incubation was continued for another 48 hours by adding DDP at the following concentrations: 0.1, 0.2, 0.4, 0.8, 1.6, and 3.2 μg/ml. Three replicate wells were created for each concentration. The medium was discarded, and then 50 μL of MTT solution was added to each well. The incubation was continued for 4 hours, and after that, the supernatant was discarded. 150 μL of dimethylsulfoxide was then added to each well with a slight shock for 5 minutes. The optical densities (OD) were measured by an enzyme counter at the wavelength of 490 nm to calculate the cell survival rate. The GraphPad Prism 8 software was used to calculate the half inhibitory concentration (IC_50_) value followed by the calculation of the resistance index using IC_50_.

The MKN45/DDP cell culture was performed as described above. The EM extract was added to the wells at low, medium, and high concentrations of 0.125 μg/mL, 0.25 μg/mL, and 0.5 μg/mL respectively while control and blank wells were set up as previously described. The OD of each group was determined after 48 hours of culture. Histograms were plotted with the survival rate plotted on the y-axis and the drug concentration on the x-axis. The IC_50_ values were then determined.

Cell survival = (experimental OD − blank OD)/(control OD − blank OD). Resistance index = IC_50_ of MKN45/DDP cells/IC_50_ of MKN45 cells.

### Assay for cell migration and invasion

MKN45/DDP cells in logarithmic growth phase were taken and Matrigel matrix gel was diluted to 300 μg/mL with serum-free cell culture medium at 4°C, and 100 μl was taken and evenly applied to the surface of the pc membrane of the cell culture cell, and then the cell was gently placed in a 24-well plate and left at 37°C for about 3 hours. Next, we added 600 μl of medium with 10% fetal bovine serum to the lower chamber, and 100 μl of cell suspension to the upper chamber. These samples were then incubated in a cell culture incubator for 48 hours. Following that, we immersed the lower surface in a 70% methanol solution and fixed it for 30 minutes. Finally, we stained the sample with Tepan blue and examined it under a microscope. We counted the number of cells on the lower surface of the membrane. The Transwell assay protocol for cell migration is identical to that of the Transwell assay for cell invasion, except that the addition of matrix gel in the upper chamber is unnecessary.

### Apoptosis assay

MKN45/DDP cells were homogeneously inoculated in 6-well plates at 5 × 10^5^ cells/well, 2500 μL. The cells were treated with a medium containing 1.2 μg/mL of DDP and 0.25 μg/mL of EM for a duration of 48 h. After treatment, the cells were harvested and washed 3 times with phosphate-buffered saline (PBS). 500 μL of 1× Binding buffer was added to the cells, and the mixture was incubated with 5 μL of Annexin V and PI dye for 15 min in the dark. The apoptotic rate of the cells was measured using flow cytometry.

### ROS experiment

MKN45/DDP cells were inoculated in 6-well plates at 1 × 10^6^ cells/well, 2500 μL. After routine trypsin digestion and centrifugation, cells were collected and set aside. A solution for staining was prepared: To achieve a final concentration of 10 μmol/L, DCFH-DA was diluted with serum-free medium in a 1:1000 ratio. DCFH-DA reached a final concentration of 10 μmol/L. Loading the probe: Staining solution was added to each sample tube at a volume of 1 mL. The samples were incubated at 37°C for 20 minutes in a cell culture incubator while being mixed upside down every 3–5 minutes to ensure full contact between the probe and the cells. The cells were collected by centrifugation and washed thrice after the incubation period. The cells were resuspended by adding 200 μL of PBS. The precipitate was blown up and mixed. The cells were then analyzed using flow cytometry, using an excitation wavelength of 488 nm and an emission wavelength of 525 ± 20 nm. The cellular ROS levels were analyzed using FlowJo software.

### LPO experiment

MKN45/DDP cells were harvested in the logarithmic growth phase. They were inoculated into 6-well plates at 5 × 10^5^ cells/well in 2500 μL. Subsequently, the cells were divided into control, EM, DDP, and combined group following surface attachment. We added various drugs to each group for continuous intervention lasting 48 hours. We collected the cells via centrifugation and performed an LPO assay using the corresponding kit’s instructions. Finally, we determined the OD value of each well at the 586 nm wavelength of the enzyme marker.

### Detection of Fe^2+^ levels

Cells were inoculated into six-well plates at a concentration of 5 × 10^5^ cells/well in 2500 μL. The cells were then treated in the groups as described above. The stock solution was prepared by dissolving 50 μg of RhoNox-1 in 110 μL of anhydrous DMSO, resulting in a 1 mM stock solution. The working solution was prepared as follows: Dilute the stock solution using PBS and prepare a working solution of RhoNox-1 at a concentration of 5 μM. The adherent MKN45/DDP cells were cultivated on sterile coverslips, which were then removed from the medium, and the excess medium was aspirated. Then, a 100 μL dye working solution was added, gently shaken to cover the cells completely, and incubated at room temperature for 30 minutes. The working solution was aspirated, washed 3 times with medium, and eventually viewed through a fluorescence microscope.

### The animals were modeled, grouped, and treated with drugs

MKN45/DDP cells (1 × 10^7^ cells/mL) in the logarithmic growth phase were inoculated under aseptic conditions into the right anterior axilla of nude mice (0.2 mL/each). SC for about 1 week, the nude mice were observed to form tumors, and all the nude mice were able to palpate the nodules under the skin, indicating that the subcutaneous transplantation of tumors into the nude mice with gastric cancer had been successfully prepared as a drug-resistant model. Nude mice that were successfully modeled were randomly divided into four groups, each with six mice: the control group, the EM group, the DDP group, and the combined group. We calculated equivalent doses based on the clinical dose commonly used for adults, the daily dosage, and the body surface area of the mice. We administered ip DDP (2 ug/kg) two times a week for eight consecutive weeks to nude mice in the DDP group. We gave ig EM decoction (2.6 g/kg) twice daily for 28 days to nude mice in the EM group. In the combined group, we administered both ip DDP (2 ug/kg) and ig EM decoction (2.6 g/kg). The model group was given an equal volume of saline continuously.

### Measurements of body weight and tumor volume were taken in nude mice

The experimental groups’ nude mice were weighed every three days since the initial administration of the intervention. Additionally, their tumors’ length (a) and width (b) were measured, and the tumor volume was determined using the formula (tumor volume = ab^2^/2). After the last dose, all nude mice were anesthetized using an intraperitoneal injection of 1% pentobarbital sodium at 4 mg/kg. The tumor tissues were then exfoliated intact, and the tumor volume was measured. Tumor growth curves were plotted using GraphPad Prism 8 software by using days and tumor volume as the horizontal and vertical coordinates respectively.

### Hematoxylin and eosin staining (HE)

We euthanized experimental nude mice using the methods described above. We extracted and fixed their tumor tissues in a 4% paraformaldehyde solution for 24 hours, rinsed them under running water for 12 hours, dehydrated them in 75%, 85%, 95%, and 100% ethanol, made them transparent using xylene, dipped them in wax, embedded and sliced them, and baked them. Subsequently, we observed pathological changes in the tumor tissues of the nude mice in each group under the microscope. We performed HE staining and sealed the samples using neutral tree glue.

### Immunohistochemistry (IHC)

Tumor tissue sections were kept in an oven for an hour. Following standard dewaxing, hydration, high-pressure antigenic heat repair, and sealing steps, dropwise addition of GPX4 (1:100) and FTH1 (1:100) was performed. Then, the sections were kept overnight in a 4°C refrigerator. After incubating with the secondary antibody for 20 minutes, we performed DAB color development, hematoxylin re-staining, dehydration, and sealing steps. Finally, it was observed under a microscope, and image acquisition was performed, with positive results expressing a brown or tan color.

### Real-time fluorescence quantitative polymerase chain reaction (PCR)

100 mg of tumor tissue was taken for each group and ground with liquid nitrogen. Then 1 mL of Trizol was added, mixed well through shaking, and left to stand for 10 minutes. Next, we added 0.2 ml chloroform, mixed it well, and allowed it to stand for 3 minutes. Afterward, centrifuge the solution at 4°C and 12,000 rpm for 15 minutes. The supernatant was removed, 0.5 ml of isopropanol was added, mixed, and allowed to stand on ice for 5 minutes, centrifuged as above and the supernatant was discarded. The precipitates were washed extensively with 1 mL of 75% ice ethanol, and the supernatant was discarded. RNA was dissolved with 1 mL of DEPC water after precipitation at room temperature for 20 min, and it was stored in the refrigerator at −80°C for backup. The concentration and purity of the extracted RNA were detected using a nucleic acid quantifier following total RNA extraction. Subsequently, cDNA was obtained by reverse transcription of RNA before PCR amplification was carried out. The sequence and length of the primer were shown in Table 1.

**Table 1:** Primer design.

**Gene name**	**Sequence**	**Length/bp**
P-gp	Upstream Primer: GGGATGGTCAGTGTTGATGGA	110
	Downstream Primer: GCTATCGTGGTGGCAAACAATA	
MRP6	Upstream Primer: GCCACCACGCTCTTCTGTC	149
	Downstream Primer: GCTACGGGCTTGTCACTCG	
β-actin	Upstream Primer: CATGTACGTTGCTATCCAGGC	250
	Downstream Primer: CTCCTTAATGTCACGCACGAT	

### Western blotting (WB)

100 mg of tumor tissue was collected for each group, added to the lysate, and placed on ice. Grinding beads were added to the mixture and ground in a cryo mill for 5 minutes. The mixture was then centrifuged at 4°C for 10 minutes, and the supernatant was used to determine the protein concentration by extracting it using the BCA Protein Content Kit. Finally, a sampling buffer was added to the samples, which were denatured in a boiling water bath for 10 minutes. The protein samples underwent transfer to a PVDF membrane through sodium dodecyl sulfate-polyacrylamide gel electrophoresis. Following this, the membrane was incubated for 15 minutes with a rapid containment solution, and primary antibodies were added: p65 (1:1000), p-p65 (1:1000), Snail (1:1000), E-cad (1:1000), P-gp (1:1000), MRP1 (1:1200), LRP (1:1000), PI3K (1:1000), p-PI3K (1:1000), AKT (1:1000), p-AKT (1:1000), mTOR (1:1000), p-mTOR (1:1000). Then followed by overnight incubation at 4°C. The next day, the membrane was rewarmed for one hour, washed five times, and the secondary antibody (1:5000) was added. It was incubated at room temperature for one hour. Thereafter, ECL chemiluminescent solution was added for exposure and image acquisition. The grey value of the target proteins was analyzed through ImageJ software.

### Statistical analyses

We analyzed the data using GraphPad Prism 8 software and reported the mean ± standard error. A one-way analysis of variance was used on the dates of three or four groups, and *t*-test was used on the dates of two groups. *P* < 0.05 indicated a significant difference.
